# *Vibrio cholerae* Exploits Sub-Lethal Concentrations of a Competitor-Produced Antibiotic to Avoid Toxic Interactions

**DOI:** 10.3389/fmicb.2013.00008

**Published:** 2013-01-31

**Authors:** Jason R. Graff, Stephanie R. Forschner-Dancause, Susanne Menden-Deuer, Richard A. Long, David C. Rowley

**Affiliations:** ^1^Graduate School of Oceanography, University of Rhode IslandNarragansett, RI, USA; ^2^Department of Biomedical and Pharmaceutical Sciences, College of Pharmacy, University of Rhode IslandKingston, RI, USA; ^3^Department of Biological Sciences and Marine Science Program, University of South CarolinaColumbia, SC, USA

**Keywords:** chemical ecology, antibiotic, colonization, competition, swimming behavior, *Vibrio cholerae*, andrimid, cell–cell interactions

## Abstract

*Vibrio cholerae* is a human pathogenic marine bacterium inhabiting coastal regions and is vectored into human food and water supplies via attachment to particles including detritus, phytoplankton, and zooplankton. Particle colonization by the pathogen is inhibited by an antagonistic interaction with the particle-associated Vibrionales bacterium SWAT3, a producer of the antibiotic andrimid. By analyzing the individual movement behaviors of *V. cholerae* exposed to a gradient of andrimid in a microfluidics device, we show that the pathogen has a concentration dependent avoidance response to sub-lethal concentrations of the pure antibiotic and to the metabolites produced by a growing colony of SWAT3-wild-type. This avoidance behavior includes a 25% increase in swimming speeds, 30% increase in run lengths, and a shift in the direction of the bacteria away from the andrimid source. Consequently, these behavioral shifts at low concentrations of andrimid would lead to higher diffusivity and result in the dispersion of bacteria away from the competitor and source of the antibiotic. Such alterations in motility were not elicited in response to a non-andrimid-producing SWAT3 mutant, suggesting andrimid may be a negative effector of chemotaxis for *V. cholerae*. The behavioral response of colonizing bacteria to sub-inhibitory concentrations of competitor-produced antibiotics is one mechanism that can influence microbial diversity and interspecific competition on particles, potentially affecting human health in coastal communities and element cycling in the ocean.

## Introduction

The ocean is a particle rich environment composed of colloids, submicrometer particles, and transparent organic particulate matter (Wells and Goldberg, 1991; Azam and Long, 2001; Passow, 2002; Verdugo et al., 2004). These particles, in addition to living organisms such as phytoplankton and zooplankton, create a heterogeneous environment in which motile microorganisms must navigate to locate resources and conditions that are suitable for their growth. Some marine microbes can maximize their utilization of patchy resources by employing chemotaxis to aggregate around phytoplankton cells, colloidal particles, and plumes of organic substrates (Mitchell et al., 1985; Blackburn et al., 1998; Menden-Deuer and Grünbaum, 2006; Azam and Malfatti, 2007; Stocker et al., 2008; Seymour et al., 2009). High rates of enzyme activities, incorporation of dissolved organic matter, and growth in these organic rich microenvironments are hypothesized to account for a large portion of the microbial production in the ocean (Azam and Long, 2001; Kiørboe and Jackson, 2001; Hunt et al., 2010) and has a significant influence on global element cycles (Smith et al., 1992; Zaccone et al., 2002).

Chemical cues structure many communities (Hay, 2009) but little is known regarding the influence of specific secondary metabolites on the behaviors of motile marine bacteria and how chemically mediated interactions structure marine microbial diversity on particles (Long et al., 2003; Grossart et al., 2004). In a typical 1 mm^3^ of surface seawater there are more than 1000 prokaryotes and eukaryotes, resulting in a proximity of less than 100 μm between these organisms (Azam and Malfatti, 2007). The aggregation of bacteria to a particle results in even closer proximity and increased intra- and interspecific interactions, all while altering the immediate surrounding environment as they process the organic rich substrates (Azam and Malfatti, 2007). Intraspecific interactions may include quorum sensing activities (Gram et al., 2002) while interspecific competition is evident in the high incidence of antagonistic interactions between marine bacteria, particularly those attached to particles (Long and Azam, 2001; Grossart et al., 2004). These chemically mediated interactions likely affect the microscale distributions of bacteria species (DeLong et al., 1993; Long et al., 2003). This is important because the bacteria present on particles differentially influence element cycling in the ocean due to variations in enzymatic activities (Smith et al., 1992; Martinez et al., 1996; Zaccone et al., 2002) and have implications for human health (Colwell, 1996).

Deciphering the mechanisms that drive bacterial diversity and abundance is key to mitigating the potentially harmful effects of pathogens such as *Vibrio cholerae*. Antagonism between bacteria is most often experimentally demonstrated in the laboratory through antibiotic activity. Many marine bacteria, particularly particle-attached bacteria, express antibiosis toward other isolates (Long and Azam, 2001; Grossart et al., 2004). An example of this is the relationship between the *Vibrio* SWAT3-wild-type (SWAT3-*wt*) and the human pathogen *V. cholerae* (Long et al., 2005). Both bacteria colonize particles and compete for resources in these microenvironments. Long et al. (2005) found that the particle-colonizing ability of *V. cholerae* is inhibited in the presence of SWAT3, a particle-colonizing competitor and a producer of the antibiotic andrimid. This antibiotic has been isolated from both cultures of marine (Needham et al., 1994; Long et al., 2005) and terrestrial Gammaproteobacteria (Fredenhagen et al., 1987). In biomedically relevant assays, andrimid blocks the carboxyl-transfer reaction of acetyl-CoA carboxylase (Freiberg et al., 2004) to which the andrimid producers show resistance (Liu et al., 2008) and demonstrates potent antibacterial activity against diverse bacteria of biomedical interest (Singh et al., 1997). *V. cholerae* did not colonize model agar-based particles that were previously colonized by the wild-type SWAT3 but readily colonized particles containing SWAT3-111, a SWAT3 mutant incapable of producing andrimid. While andrimid potently inhibits the growth of *V. cholerae*, it is unclear how sub-lethal concentrations could act to mediate this competitive interaction. Sub-lethal interactions are likely to be found in naturally occurring environments where it may be difficult to maintain growth-inhibiting concentrations of an antibiotic by competing species due to the effects of rapid diffusion. Exploring cell–cell interactions at more realistic, sub-lethal concentrations is important for understanding the regulation of bacterial diversity, but also because particle attachment and subsequent growth is the major mechanism for the transmission of an infective dose of *V. cholerae* to a human host (Colwell, 1996). Deciphering the mechanisms leading to particle attachment by *V. cholerae* is particularly pressing since increasing outbreaks of cholera have been attributed to changes in global climate and climatic events (Colwell, 1996).

We hypothesized that andrimid’s role in the established antagonistic interaction is to function as a chemical signal that deters *V. cholerae* from colonizing particles. To test this hypothesis, we developed a chemotaxis assay that incorporates polydimethylsiloxane (PDMS) microchannels with a disk or colony diffusion assay (Bauer et al., 1966) and quantified the swimming behaviors, including swimming speeds and turning rates, of individual motile bacteria using microvideography and cell tracking. In these experiments, the movements of individual bacteria were tracked as they swam over an agar surface containing a linear gradient of pure andrimid or the suite of metabolites produced by a growing colony of bacteria. We determined that a gradient of diffusible metabolites from SWAT3*-wt* induced concentration specific changes in the swimming behavior of *V. cholerae* that effectively, although not completely, resulted in avoidance of the toxic agent. Purified andrimid elicited a similar response, suggesting it was the principle metabolite affecting the motility and colonization behavior of *V. cholerae*.

We expected the swimming patterns of *V. cholerae* to be altered when exposed to lethal concentrations of andrimid, i.e., cells stop swimming as they succumb to the compound and the possible movement of cells away from toxic concentrations. Surprisingly, *V. cholerae* showed alterations in behavior to sub-lethal concentrations of andrimid, either the pure compound or when produced by a colony of SWAT3*-wt*, that would result in a flux of cells away from the source and out of the sub-lethal area, ultimately reducing exposure of the cells to toxic concentrations of andrimid. These results suggest that *V. cholerae* alters its swimming behavior to avoid contact with potentially lethal concentrations of an antibiotic and suggest that sub-lethal concentrations of andrimid may serve as interspecific signaling molecules in the marine environment to deter particle colonization and ultimately competition for resources.

## Materials and Methods

**Figure 1:**
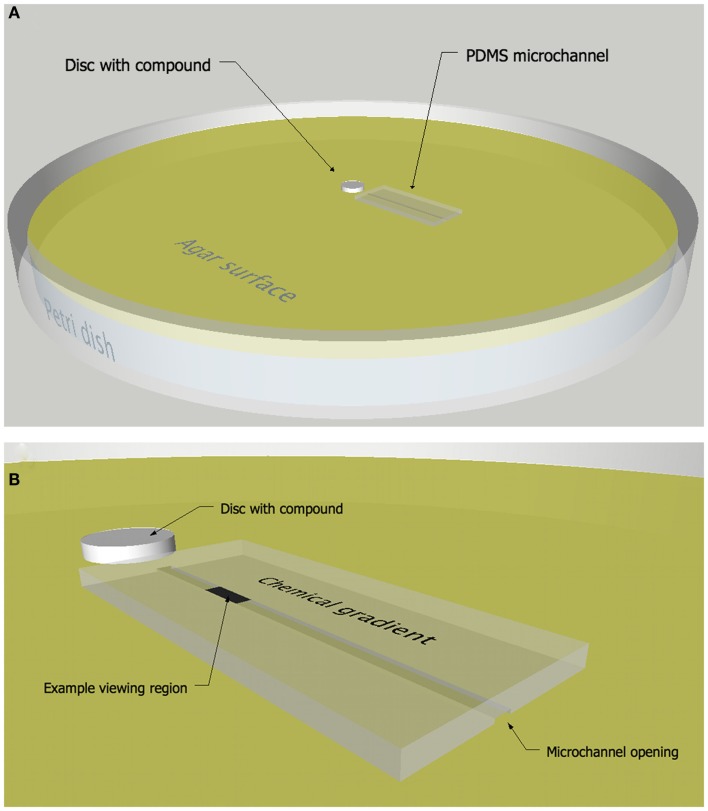
**Microchannel chemotaxis response assay**. Diagram **(A)** and close-up **(B)** of the microchannel chemotaxis assay in which a PDMS microchannel was placed onto an agar surface containing a chemical gradient of compounds diffusing from a paper disk or growing colony of bacteria.

A microchannel chemotaxis assay that combines a PDMS microchannel (Whitesides et al., 2001; Sia and Whitesides, 2003) and a disk or colony diffusion assay (Bauer et al., 1966) was developed which allows bacterial behavior to be observed and quantified in spatially structured chemical environments as they swim in close proximity to a chemically modified surface (Figure 1). Specifically, the movements of individual bacteria are tracked within a microchannel as they react to a concentration gradient of secondary metabolites supplied from an agar surface. The gradient was first established in the agar matrix by diffusion away from a disk or growing bacteria colony. The diffusion of secondary metabolites away from the source creates a chemical gradient with the highest concentration nearest the disk or colony. A PDMS microchannel composed of three walls, i.e., with one open side, is placed onto the agar plate and the agar provides the fourth wall of the channel (Figure 1). Solution from the agar fills the microchannel, creating an aquatic microenvironment suitable for motile microorganisms to move within the confines of the channel (DiLuzio et al., 2005). The channel is arranged radially outward from the disk so that the chemical gradient in the agar is aligned with the channel. Bacteria are introduced into the channel away from the chemical source and their movements are imaged at specific times and/or spatial intervals using a compound microscope and microvideography techniques. In addition to creating a chemical gradient, the diffusion assay helps define the potential regions of interest, such as a zone of growth inhibition, as they relate to the concentration of chemical signals in the agar.

### Microchannels

Microchannel templates were drawn using SolidWorks CAD software (SolidWorks Corp.) and photolithography masks from these designs were printed at 10,000 dpi resolution (CAD Art Services, Inc., Brandon, OR, USA). PDMS microchannels were fabricated at the University of Rhode Island in the Engineering Department’s Microfluidics Clean Lab using soft lithography techniques (Xia and Whitesides, 1998). Microchannels were fabricated with dimensions of ∼1.4 cm long × 400 μm wide × ∼50 μm deep.

### Bacteria cultures

Colonies of SWAT3-*wt* or SWAT3-111 were created by spotting 2 μl of cells grown overnight in ZoBell (ZB) broth (ZB: 15 g peptone, 5 g yeast extract, 15 g instant ocean, 500 ml deionized H_2_0) at 24°C onto the center of a ZB/8 (1/8 nutrients of ZB) agar plate. The colonies were grown for 16–20 h at 24°C. *V. cholerae* N16961 was cultured overnight in ZB broth at 24°C and shaken at 175 rpm.

### Growth inhibition and minimum inhibitory concentration of andrimid in agar

To assess the zone of growth inhibition due to the presence of pure andrimid or to metabolites produced by colonies of SWAT3-*wt* and mutant SWAT3-111, we carried out disk (125 μg andrimid disk^−1^) and growing colony diffusion assays. We also determined the minimum inhibitory concentration (MIC) of andrimid using Kirby–Bauer disk diffusion assays (Bauer et al., 1966). For this assay, 10 μl of andrimid dissolved in methanol, ranging in concentration from 3.75 ng ml^−1^ to 1.835 μg ml^−1^, were loaded onto a sterile 6 mm paper disk and allowed to air dry. *V. cholerae* was diluted to the equivalent of a 0.5 McFarland standard (% transmittance = 74.3, absorbance at 600 nm = 0.132) and inoculated onto the surface of 8.5 cm diameter agar plates containing 15 ml of ZB/8 media. A single disk was placed onto the center of the plate and incubated at 24°C. The diameter of the zone of growth inhibition of *V. cholerae* was measured after 18 h. The zone diameters and concentrations for disk loadings were used to estimate the MIC using a linear regression model (Bonev et al., 2009).

### *Vibrio cholerae* chemotaxis assays

To determine if andrimid influences the swimming behavior of *V. cholerae*, assays were performed with pure andrimid and colonies of SWAT3-*wt*, an andrimid-producing competitor of *V. cholerae*. For the pure andrimid assays, 125 μg of the pure compound, which provided a zone of growth inhibition similar in size to the growing colony of SWAT3, were loaded onto a sterile paper disk and placed into the center of a ZB/8 agar plate for 16–20 h prior to running chemotaxis assays. For the colony assay, 2 μl of SWAT3-*wt* growing in ZB media were spotted onto the center of a ZB/8 agar plate and incubated at 24°C for 16–20 h. These concentrations and volumes were identical to the disk and colony diffusion assays used to assess growth inhibition due to the presence of pure andrimid or SWAT3 secondary metabolites. Control assays were also performed using SWAT3-111, an andrimid knockout mutant (Long et al., 2005), and agar plates without andrimid but supplied with a paper disk, loaded with methanol and dried prior to use, the same procedure and carrier used to load andrimid onto paper disks.

To introduce *V. cholerae* into the channel, a 1 ml sub-sample of culture was centrifuged at 7000 rpm for 3 min, the supernatant removed, and the cells re-suspended in sterile filtered seawater media. A 1 μl aliquot of the re-suspended cells was then spotted near the microchannel opening and the seawater media was absorbed into the agar. Just prior to video capture, the microchannel was gently maneuvered toward the *V. cholerae* that were pipetted onto the agar until motile cells were visible at the channel entrance.

Once inside the channel, cells began moving away from the channel entrance and up the chemical gradient. Motile bacteria were imaged in a 200 μm × 460 μm region in the center of the microchannel at 1 mm distances from the edge of the disk, colony, or other region of interest, e.g., the edge of a zone of growth inhibition. A Nikon Eclipse TE2000-E inverted microscope with an automated stage was used to position and capture images within the microchannel. Images were acquired at 20× magnification for 60 s at a minimum of 15 frames per second (fps) using a Photometrics CoolSNAP HQ camera and Nikon Elements v. 3.0 software. A minimum of two replicates for each experiment was performed and data from identical distances from the chemical source were collected and pooled to increase the number of observations at each distance. Three zones were identified within the treatment data. These zones were defined based upon a known region that would limit growth or *post hoc* from behavioral observations. Operationally, areas of the channel closest to the andrimid source and overlying agar surfaces within the experimentally determined zone of growth inhibition were defined as the lethal zone. The section of the channel extending 4.5 mm beyond the lethal zone was defined as the sub-lethal zone based upon alterations in behavior when compared to control values. The region furthest from the andrimid source where bacteria exhibited behavior similar to that of control values was termed the non-lethal zone. While these zones do not distinguish the more gradual behavioral responses with respect to a continual chemical gradient, they were chosen to highlight statistically unique regions within the channel.

### Image processing and data analysis

The 60 s movies, captured at >15 fps, were manually processed to determine the image threshold, which was applied to automatically remove background noise and determine the Cartesian coordinates of bacteria in each video using Image J (NIH). Swimming tracks of the bacteria were assembled from 2D positions using a 2D version of an automated 3D tracking program (Menden-Deuer and Grünbaum, 2006). High frequency noise was removed from paths with a cubic smoothing spline and swimming statistics were sampled from these paths at 0.05 s intervals. The methods for organism tracking closely follow established methods for tracking marine protists (Menden-Deuer and Grünbaum, 2006). Only cells tracked for a minimum of 1 s were included in this analysis. Multiple components of swimming behavior including the swimming speed, velocity, turning rate, run length, and path direction, were calculated using the software MATLAB v 7.4. This analysis provided hundreds of swimming trajectories and upwards of tens of thousands of movement statistics per treatment (*n* > 950,000 for the entire experiment for a single swimming behavior) to characterize the bacterial behaviors as a function of metabolite exposure and within channel position. See Presentation S1 in Supplementary Material with videos for an example of this process.

### Andrimid production and purification

Andrimid was produced and purified as previously described (Long et al., 2005). Briefly, SWAT3-*wt* was cultured in ZB 2216 medium at 23°C on a rotary shaker for 4 days. Ethyl acetate extraction of the whole culture broths yielded extracts with potent growth inhibitory activity against *V. cholerae*. Purification of andrimid was achieved by bioassay-guided fractionation using column chromatography [Amberchrom CG-161m; stepwise gradient of methanol (MeOH) in water] and reverse-phase high-pressure liquid chromatography (HPLC; 45–80% MeOH in H_2_O over 20 min at 10 ml min^−1^; Waters Xterra RP18 5 μm, 19 mm × 100 mm column). Pure andrimid was verified by ^1^H and ^13^C NMR spectroscopic data and mass spectrometry measurements in comparison with literature values (Fredenhagen et al., 1987).

### Statistical analysis

To compare behavioral responses to the different metabolite stimuli presented, the non-parametric Kolmogorov–Smirnov test was used to compare the distributions of movement statistics from control and treatment assays and among the various distances within each treatment. Statistical significance was assigned when *p* < 0.05.

## Results

### *Vibrio cholerae*’s behavior in control assays

Microchannel control assays were performed on (1) ZB/8 agar plates (growth media with no chemical stimuli) or (2) with a growing colony of SWAT3-111, a SWAT3 andrimid knockout mutant. For both controls, movement behaviors were not related to position in the channel, indicating that the location within the channel or metabolites produced by the mutant had no effect on organism motility. Thus, data collected from all positions in the channel were aggregated to represent each control. The mean behaviors were very similar for the swimming speeds (52.6 and 53.2 μm s^−1^), turning rates (100° s^−1^ and 108° s^−1^), and run lengths (19.9 and 20.1 μm) for the media blank and SWAT3-111 control, respectively (Table 1).

**Table 1:** **Mean swimming behaviors for each control and for each defined zone for each treatment**.

Control/treatment	Speed (μm s^−1^; SE)	Turning rate (° s^−1^; SE)	Run length (μm; SE)	Total no. of observations	Number of replicates
Media blank	52.6 (0.05)	100 (0.43)	19.9 (0.18)	204,175	20
SWAT3-111	53.2 (0.08)	108 (0.64)	20.1 (0.23)	101,297	15
**ANDRIMID (ZONE)**					
Non-lethal	55.8 (0.09)	115 (0.76)	19.1 (0.23)	78,311	14
Sub-lethal	63.7 (0.17)	107 (1.17)	25.2 (0.46)	27,230	5
Lethal	44.3 (0.07)	117 (0.64)	16.3 (0.18)	101,845	12
**SWAT3-wt (ZONE)**					
Non-lethal	50.3 (0.12)	111 (0.95)	20.4 (0.35)	44,126	4
Sub-lethal	60.1 (0.05)	112 (0.38)	22.1 (0.14)	264,842	28
Lethal	43.2 (0.06)	118 (0.52)	15.2 (0.14)	146,750	9

*SE, standard error of the mean, calculated as the standard deviation divided by the square root of the number of observations*.

### *Vibrio cholerae* alters its motility in response to andrimid and SWAT3-*wt* metabolites

**Figure 2:**
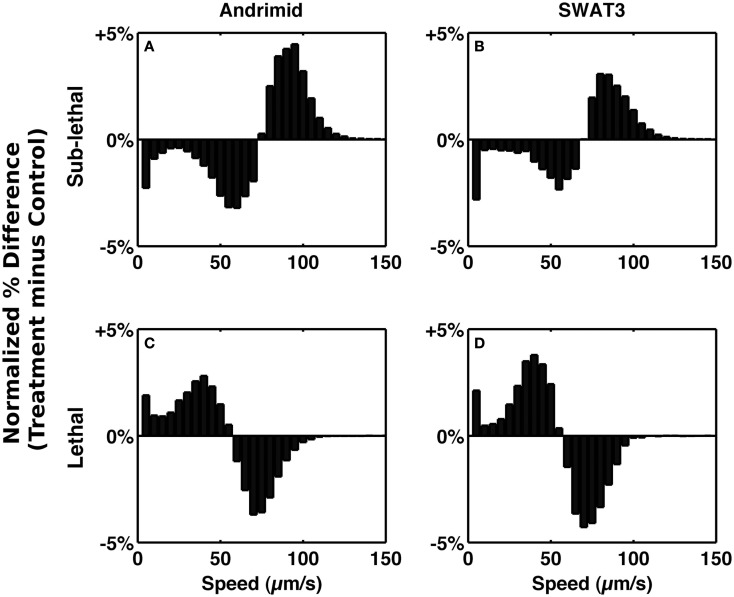
**Alteration of speed frequency**. Bars represent the normalized differences between treatment and control speed frequency distributions in the sub-lethal zone **(A,B)** and lethal zone **(C,D)**. **(A,C)** Andrimid treatment minus media control. **(B,D)** SWAT3 minus SWAT3-111 control.

**Figure 3:**
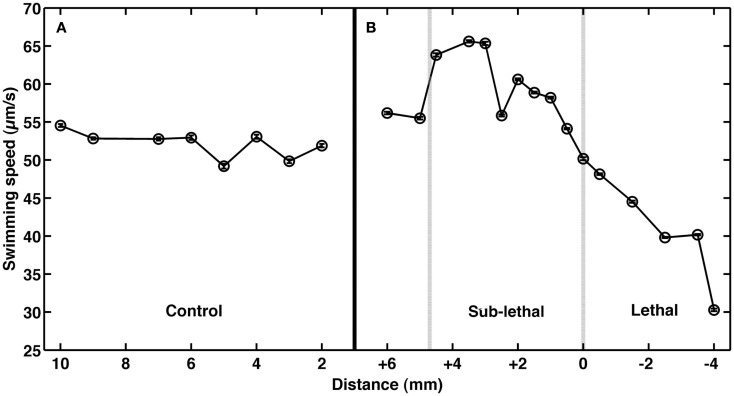
**Impact of andrimid on *Vibrio cholerae* swimming speed**. Mean swimming speeds of *V. cholerae* at distances **(A)** from a colony of SWAT3-111 (control) and **(B)** inside the sub-lethal (+) and lethal (−) zones where 0 mm indicates the delineation of the zone of growth inhibition created by an andrimid-producing colony of wild-type SWAT3. Swimming speeds increase by >10% in the sub-lethal zone and decrease upon exposure to lethal concentrations of andrimid. Plotted error bars are contained within data symbols and represent 1 standard error of the mean.

To test the role of andrimid in eliciting *V. cholerae* behavioral response, the pathogen was exposed to two andrimid gradients: (1) the metabolites produced by a colony of SWAT3-*wt*, and (2) pure andrimid. In the non-lethal or distal zone from the source (i.e., disk with andrimid or SWAT3 colony) *V. cholerae’s* swimming speeds, turning rates, and run lengths were similar to values observed in the controls (SWAT3-111 and agar; Table 1). This suggests that *V. cholerae* is under little or no influence of andrimid or other bacterial metabolites in the non-lethal region. In contrast, *V. cholerae* significantly altered its swimming behavior in the sub-lethal zone. Mean swimming speeds for all observations within the sub-lethal zone for the andrimid and SWAT3-*wt* assays were 63.7 and 60.1 μm s^−1^ (Table 1), an increase of ∼10% from the mean swimming speeds of 55.8 and 50.3 μm s^−1^ in the non-lethal zone. Within the sub-lethal zone, 23% more of the measured speeds exceeded 70 μm s^−1^ (Figures 2A,B) in comparison to controls. The highest mean swimming speeds, observed at any single distance from the stimulus, were recorded within the sub-lethal zones of the andrimid and SWAT3-*wt* treatments, reaching 67.4 and 65.6 μm s^−1^, respectively; an increase of 28 and 23% in comparison to the controls. Bacteria observed within the lethal zone had slow swimming speeds, with 18% more of the observations less than 55 μm s^−1^ (Figures 2C,D) when compared to control data. Swimming speeds for cells inside the lethal zone averaged 43.2 μm s^−1^ for the SWAT3-*wt* colony and 44.3 μm s^−1^ for the andrimid treatments (Table 1). Average swimming speeds decreased by more than 30 and 40% to 30.2 and 36.6 μm s^−1^ for cells swimming 4 mm inside the lethal zone created by the SWAT3*-wt* colony and the andrimid, respectively. The progression and changes in *V. cholerae’s* swimming speed as the bacteria traveled up the gradient of andrimid produced by SWAT3-*wt* were observed in the data for specific distances from the disk or colony within each of these zones (Figure 3).

In both the SWAT3 and andrimid treatments, other parameters of motility were affected in addition to speed. Bacteria turning rates in the sub-lethal zone were 107° s^−1^ and 112°s^−1^ and were greatest in the lethal zones, increasing to 117° s^−1^ and 118°s^−1^ (Table 1). Run lengths, in comparison to the control assays, were longest in sub-lethal zones at 25.2 and 22.1 μm and shortest in lethal zones at 16.3 and 15.2 μm, for andrimid and SWAT3 treatments (Table 1).

### Rate of bacterial diffusion and direction of swimming indicates an aversion to potentially lethal zones

Bacterial motility is frequently modeled as a random walk so that population distribution, and changes therein can be approximated similar to the diffusion of molecules (Berg, 1993). Measured swimming speeds and run times of tracked bacteria were used to calculate the treatment-specific bacterial diffusion coefficient, which is calculated as *D* = (*v*^2^τ)/3, where *D* = diffusivity (μm^2^ s^−1^), *v* = speed (μm s^−1^), and τ = run duration (s; Berg, 1993). A high diffusivity implies a high rate of population dispersal, caused by bacteria swimming with high speeds and low turning rates for example, to disperse from a point in space. A behavioral shift toward lower diffusivity, effected through lower swimming speeds or higher turning rates, or both, implies that the population will have a lower dispersal rate and may aggregate at a source of a chemoattractant (Berg, 1993; Packer and Armitage, 1994). We calculated the diffusivity for *V. cholerae* in our experiments using the mean swimming speeds and run durations from each treatment and zone of interest. The diffusion coefficient for the media and SWAT3-111 controls were 320 (±2.6 Standard Error of the Mean, SE) and 324 (±3.7 SE) μm^2^ s^−1^, respectively (Table 2). In the sub-lethal zone, the diffusion coefficient of *V. cholerae* was 25% higher, increasing to 417 (±7.5 SE) and 426 (±2.5 SE) μm^2^ s^−1^. In the lethal zone, this decreased to 224 (±2.5 SE) and 206 (±1.8 SE) μm^2^ s^−1^, 31 and 39% lower, respectively.

**Table 2:** **Calculated diffusion coefficients (±1 compounded standard error of the mean, SE) for *V. cholerae***.

Treatment		Diffusion coefficient (μm s^−1^; ±SE)
Andrimid	Media control	320 (2.6)
	Sub-lethal zone	417 (7.5)
	Lethal zone	224 (2.5)
SWAT3-*wt*	SWAT3-111 control	324 (3.7)
	Sub-lethal zone	426 (2.5)
	Lethal zone	206 (1.8)

**Figure 4:**
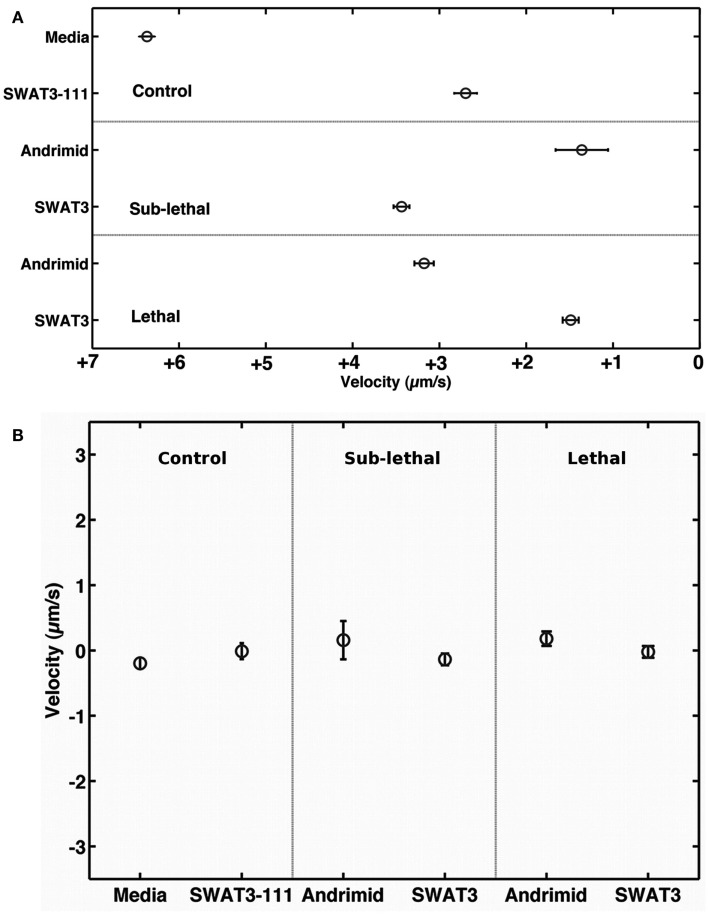
**Along and across gradient velocities**. Mean velocity of *V. cholerae* in **(A)** the down and **(B)** cross-channel direction, i.e., parallel and perpendicular to the chemical gradient. Error bars represent 1 standard error of the mean.

**Figure 5:**
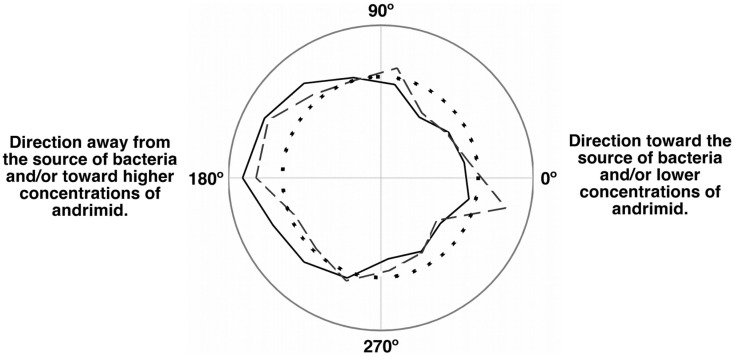
**Population momentum in response to an andrimid gradient indicated avoidance behavior**. Circular frequency distribution of the net swimming direction for the media control (solid), andrimid treatment (dash), and random distribution (dots). Zero degree is the location of the microchannel entrance and 180° is the location of the compound source. In the media control, cells moved preferably down-channel and away from the point of introduction. In the andrimid treatments, a higher proportion of paths were directed toward lower andrimid concentrations and away from the source of the metabolite.

The net velocity of *V. cholerae* was a function of the chemical gradient to which it was exposed. Irrespective of treatment, large differences in net velocity occurred in the along channel direction, parallel to the chemical gradient (Figure 4). In these assays, positive velocities indicate movement toward the far end of the channel and stimulus source. Less positive values would indicate movement toward the point of introduction of the bacteria. The average net velocity of *V. cholerae* in the media control was +6.4 μm s^−1^. Movement toward the stimulus source (i.e., positive net-velocities) were also measured for *V. cholerae* in all other experiments, but was reduced by >50% in comparison to the media control (Figure 4A). In contrast, average net-velocities in the cross-channel direction, i.e., perpendicular to the stimulus gradient, were ∼0 μm s^−1^ for all controls and treatments (Figure 4B). The swimming directions of *V. cholerae* cells were also altered in response to andrimid. Figure 5 is a circular frequency distribution of swimming trajectories. The media control (solid) and all tracks for the andrimid treatment (dash) where 180° represents the direction of the metabolite source and 0° is the direction of the channel entrance where bacteria were introduced into the channel. There was a significant shift in trajectory between the media control and the pure andrimid treatment data (Figure 5, KS-test, *p* = 0.0006) but not when comparing the SWAT3-111 control and SWAT3*-wt* assays (KS-test, *p* = 0.18, data not shown).

### Andrimid inhibits the growth of *V. cholerae*

Growth of *V. cholerae* was not inhibited on a ZB/8 agar plate or in the presence a mutant SWAT3-111 colony. In contrast, *V. cholerae* did not proliferate in a region 1.2 cm from the edge of the disks loaded with andrimid (125 μg disk^−1^) or 1 cm from the edge of a colony of SWAT3-*wt*. These distances were used to determine the placement of the microchannel in subsequent microchannel chemotaxis experiments.

The concentration of andrimid at the distal edge of the zone of growth inhibition for the disk diffusion assays was estimated to be ∼63 nM. This was calculated using a linear diffusion model for assessing the MIC of antibiotics in disk agar diffusion assays (Bonev et al., 2009). This result is in close agreement with a previous study that reported a 50% inhibitory concentration of 80 nM for andrimid against the same strain of *V. cholerae* using broth dilution assays (Long et al., 2005).

## Discussion

A lot of research is dedicated to the chemotaxis of bacteria, most notably *Escherichia coli* (Berg and Brown, 1972; Baker et al., 2005). However, little is known about species-specific chemically mediated interactions among marine bacteria, including reactions to competitor-produced secondary metabolites. Our analysis revealed that *V. cholerae* exhibited a response to sub-lethal concentrations of andrimid by increasing its swimming speed, turning rate, and run lengths and directing its movements away from the metabolite source. This competitor-induced modulation of movements effectively is negative chemotaxis and results in decreased exposure of *V. cholerae* to higher concentrations of the competitor-derived effector. These results suggest a mechanism by which *V. cholerae* exploits competitor-derived chemical information to limit exposure to the lethal antibiotic. Long et al. (2005) observed decreased attachment by *V. cholerae* to particles previously colonized by SWAT3. Our results provide a mechanistic explanation of these observations. Ultimately, these types of avoidance behaviors will alter the rates of particle attachment by the pathogen, and modify its trajectory through the food web and thus the extent of human exposure.

**Figure 6:**
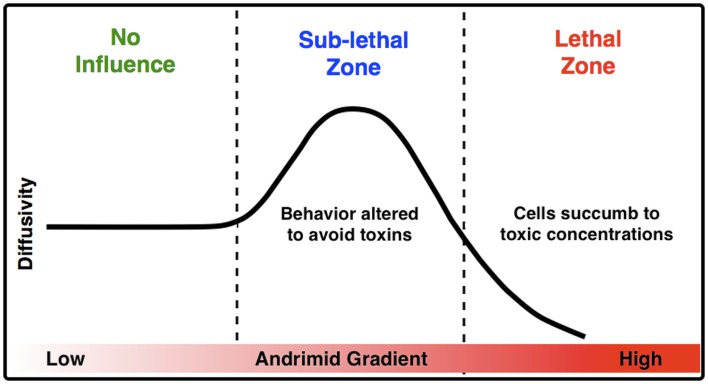
**A conceptual model for the impact of inhibitory molecules on bacteria about a point source**. Based upon empirical measurements of chemically modulated *V. cholerae*’s diffusivity (μm^2^ s^−1^) in a gradient of andrimid.

The measured increase in bacterial diffusivity and the change in the direction and velocity of cells when exposed to andrimid strongly suggest that one mechanism for limiting *V. cholerae*’s colonization on particles already colonized by SWAT3-*wt* (Long et al., 2005) is through behavioral modification and potentially sub-lethal interactions. Cells of *V. cholerae* nearing a particle colonized by the andrimid-producing SWAT3-*wt* would modify their swimming in a manner consistent with avoidance behavior. In a conceptual model for the influence of andrimid upon the behavior of bacteria susceptible to this antibiotic, and particularly *V. cholerae* (Figure 6), as a population of bacteria approaches a source of andrimid, they are initially in a zone of no influence or possibly at concentrations below their detection limit. Moving closer to the andrimid source, the cells reach sub-lethal concentrations that induce behavioral shifts leading to higher population dispersal rates as a result of increased swimming speeds and lower turn rates with a significant shift in preferred movement direction away from the source of metabolites. These altered swimming behaviors would allow a higher percentage of cells to avoid potentially toxic environments. Lastly, cells that continue swimming toward the antibiotic source will encounter lethal concentrations of the metabolite. If exposed long enough, the bacteria would succumb to the toxic effects of andrimid. Therefore, the observed avoidance behaviors should be under a strong selective pressure. It is most likely that the 40% speed reduction observed in the lethal zone was a result of the negative effect the antibiotic has on the cells. Ultimately, cells were trapped within this zone, indicated by a low population dispersal rate leading to cell death.

The continuation of *V. cholerae* up a gradient of andrimid and into potentially toxic concentrations was unavoidable in this assay. Considering the inherently diffusive nature of motile bacteria, it is inevitable that some portion of the cells would continue to be displaced toward the toxic point source, albeit at a lower rate in the presence of andrimid. These cells would ultimately perish when exposed to such high concentrations of andrimid as their motility becomes compromised, rendering cells unable to leave the toxic region. The channel may have enhanced the progression of *V. cholerae* up this gradient, as cells were ultimately unable to move beyond its confines and further away from the andrimid source and diffusion carried a portion of the population into the lethal zone. While the lowest rates of bacterial diffusion were observed within the zones of the microchannels exposing the cells to lethal concentrations of andrimid, this result is likely due to a toxic reaction and not the aggregation of cells to a particular site for colonization. Other researchers have observed the avoidance of toxins or repellents in microfluidics chemotaxis assays (Englert et al., 2009). Our observation that *V. cholerae* continued to swim into an otherwise hostile environment is not unique. Bacteria have also been observed to swarm into regions of agar plates containing toxic concentrations of antibiotics (Butler et al., 2010). Such unexpected results can complicate the interpretation of chemotaxis experiments and bacterial behavior. Additional direct observations and measurements of bacterial responses to antagonistic interactions are important to gain new insights into how microbes interact with the natural world and why they would move into potentially lethal environments.

Antibiotics are best known for lethal activity against target bacteria. We observed that andrimid, a secondary metabolite and antibiotic produced by *Vibrio* sp. SWAT3, modulates the behavior of *V. cholerae* at sub-lethal concentrations. Thus, competitor-derived antibiotics can function as an infochemical that can be exploited, effectively altering the distribution and colonization rate of *V. cholerae*. This research supports a growing ideological framework in which antibiotics are thought to be signaling molecules and serve roles other than antibiosis in the environment (Davies et al., 2006; Linares et al., 2006; Mlot, 2009). While antibiotics at high concentration have toxic effects on competitors, as is often demonstrated in the laboratory, they may also function at sub-lethal concentrations to regulate gene expression, induce biofilm formation, and alter motile responses in bacteria (Linares et al., 2006). The chemotaxis response of Vibrios has been associated with their ability to form biofilms and establish symbiosis and pathogenesis (Bulter and Camilli, 2005). As demonstrated here, andrimid likely plays a larger role than as a potent antibiotic (Freiberg et al., 2004). At sub-lethal concentrations, andrimid and other antibiotics may be interspecific signaling molecules with important ecological consequences, providing advanced warning to potentially toxic environments and deterring resource competition. This study is the first to provide evidence that andrimid acts as a signaling molecule at sub-lethal concentrations.

Interactions taking place on or near nutrient rich particles likely determine the winners in the competition for space and resources and ultimately the abundance of potential pathogens in the environment. The concentration of antibiotics is unlikely to be maintained at lethal concentrations in the diffusive marine environment. Sub-lethal concentrations near competitive surfaces are more likely to be experienced by target organisms. In addition, non-lethal interactions are also taking place in these environments. In our experiments, the metabolites from SWAT3-111, the non-andrimid-producing strain, also influenced the behavior of *V. cholerae*; resulting in similar mean net-velocities in the direction parallel to the chemical gradient. Non-lethal interactions such as these are even less understood than the more obvious and lethal relationships more commonly observed and studied in growth competition assays. Such chemically mediated cell–cell interactions, have direct implications for elemental cycling in the ocean (Smith et al., 1992; Martinez et al., 1996; Long et al., 2003) as well as the spread or outbreak of diseases (Tamplin et al., 1990; Pruzzo et al., 2008) and the occurrence, toxicity, and termination of harmful algal blooms in coastal regions (Kim et al., 1998; Alavi et al., 2001; Bates et al., 2004) where colonizing bacteria are implicated. Thus, chemically mediated microbial interactions have potential downstream influences on large scale regional and global processes. The results presented here, and studies like it (Blackburn et al., 1998; Long et al., 2003; Menden-Deuer and Grünbaum, 2006; Stocker et al., 2008; Seymour et al., 2009), are shedding new light on microorganism behaviors and the mechanisms behind cell–cell interactions in marine microenvironments.

## Conflict of Interest Statement

The authors declare that the research was conducted in the absence of any commercial or financial relationships that could be construed as a potential conflict of interest.

## Supplementary Material

The Supplementary Material for this article can be found online at http://www.frontiersin.org/Aquatic_Microbiology/10.3389/fmicb.2013.00008/abstract
